# Between land and sea

**Published:** 2018-03-27

**Authors:** Lucy Smith

**Affiliations:** 1 Memorial University of Newfoundland, Newfoundland, Canada

**Figure:**
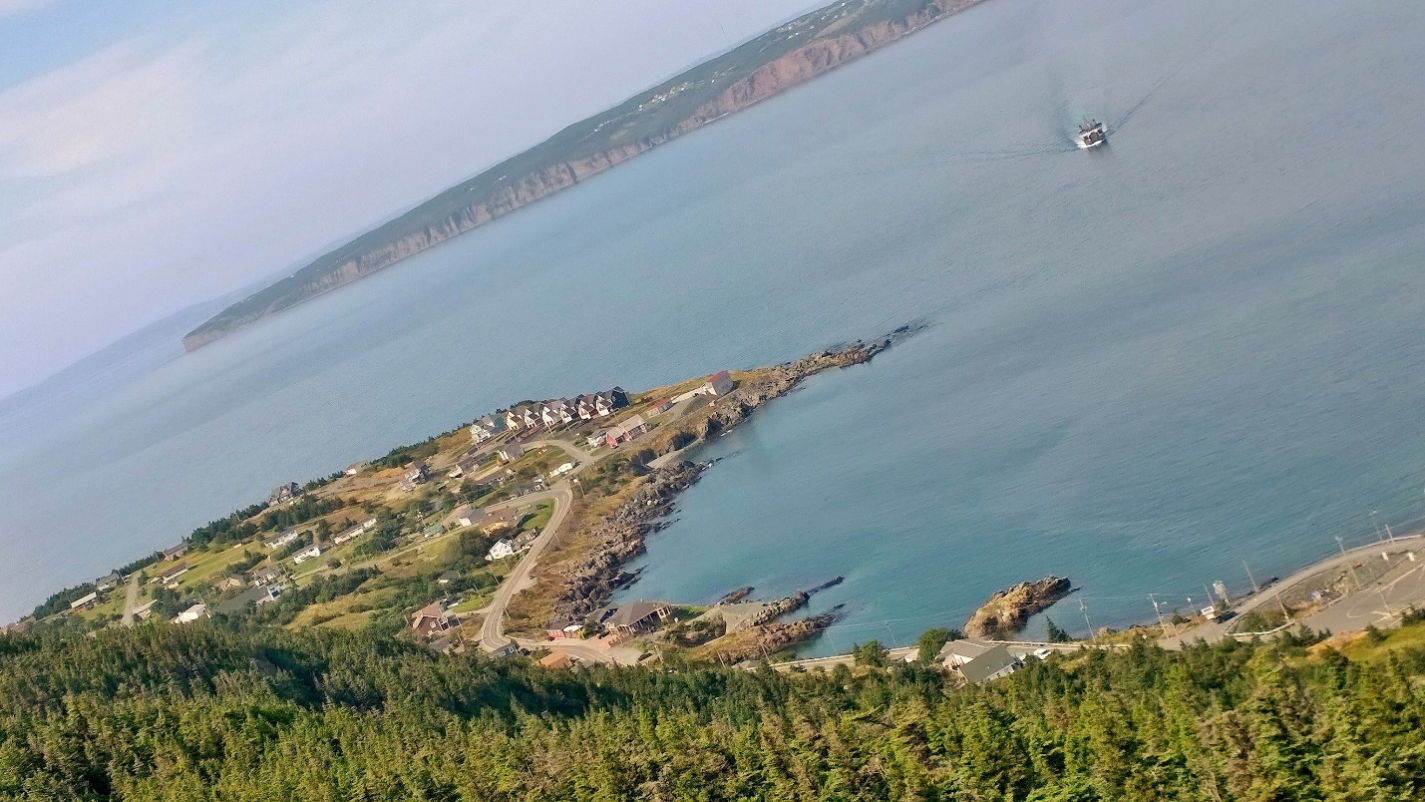


**Location**: Portugal Cove-St. Philip’s, Newfoundland and Labrador

Photo taken from the top of Grayman’s Beard, a large bed of rocks overlooking Portugal Cove at the top of a 500-foot hill.

This photograph shows the ferry from Bell Island coming into Portugal Cove. Bell Island is an island community located off the Avalon Peninsula of Newfoundland and Labrador. Residents of Bell Island rely on ferry crossing to Portugal Cove and its nearby city of St. John’s for healthcare. This symbolically illustrates the challenges of social and physical isolation of Newfoundland from the mainland of Canada.

Although the ferry crossing itself only takes 20 minutes each way, in order for a family with a small infant to be at a 10am doctor’s appointment in St. John’s, they would have to wake up at 5am to get ready and wait for the ferry. This family with a newborn baby from Bell Island repeatedly expressed how grateful they are to be able to see a family doctor in Portugal Cove instead of having to travel all the way to St. John’s for medical care. The physical isolation, lack of accessible health care services in addition to income, job as well as food insecurity are all major challenges faced by the residents of Bell Island. In comparison to Portugal Cove-St. Philip’s, the population health of Bell Island residents are at a significant disadvantage due to all the negative social, physical and environmental factors. In a symbolic sense, the Bell Island and Portugal Cove situation illustrates the reliance of Newfoundland on the mainland of Canada. The population health of our province faces many of the same social, economic and physical challenges endured by the residents of Bell Island. With a decline of the fishery and oil industry, Newfoundland and Labrador’s population health is at increased risk of food and job insecurity in addition to the physical isolation.

